# The Cervical Osteology of *Okapia johnstoni* and *Giraffa camelopardalis*

**DOI:** 10.1371/journal.pone.0136552

**Published:** 2015-08-24

**Authors:** Melinda Danowitz, Nikos Solounias

**Affiliations:** 1 Department of Anatomy, New York College of Osteopathic Medicine of New York Institute of Technology, Old Westbury, New York, United States of America; Sichuan University, CHINA

## Abstract

Giraffidae is the only family of ruminants that is represented by two extant species; *Okapia johnstoni* and *Giraffa camelopardalis*. Of these taxa, *O*. *johnstoni* represents a typical short-necked ungulate, and *G*. *camelopardalis* exemplifies the most extreme cervical elongation seen in any ruminant. We utilize these two species to provide a comprehensive anatomic description of the cervical vertebrae. In addition, we compare the serial morphologic characteristics of the okapi and giraffe cervical vertebrae, and report on several osteologic differences seen between the two taxa. The giraffe neck appears to exhibit homogenization of C3-C7; the position of the dorsal tubercle, thickness of the cranial articular process, shape of the ventral vertebral body, and orientation of the ventral tubercle are constant throughout these vertebrae, whereas these features are serially variable in the okapi. We also report on several specializations of the giraffe C7, which we believe relates to an atypical cervico-thoracic junction, corresponding to the substantial neck lengthening. The morphologic differences exhibited between the okapi and giraffe cervical vertebrae have implications on the function of the necks relating to both fighting and feeding.

## Introduction

The elongated giraffe neck clearly stands out as one of the most notable evolutionary adaptations in mammals. While general morphologic studies have been done, the detailed anatomic features of the remarkable neck have yet to be adequately studied. The vertebral length and general osteologic features of the giraffe cervicals have been compared to other extant ungulates, including the okapi [1,2]. Two studies have focused on the posterior cervical vertebrae, which concentrated on the atypical cervico-thoracic junction and the brachial plexus [3,4]. A comprehensive study of the functional anatomy of any ruminant neck would be complicated, as it would involve complete dissection of approximately 36 muscle types, kinematic studies, and biomechanical analyses. The osteology of the cervical vertebrae is an essential step in the evaluation of the structure and function of the neck, and facilitates future research. The giraffe and the okapi are the only extant members of a previously species-rich family with approximately 25 members [5]. An osteologic study of the giraffe and the okapi is ideal to provide a comprehensive analysis of the ruminant cervical vertebrae; the okapi is representative of a short-necked ungulate and the giraffe exemplifies the most extreme cervical elongation.

It has been proposed that the giraffe possesses 8 cervical vertebrae due to the morphologic similarities between the giraffe T1 and a typical ruminant C7 [3]. Evidence on the phylogenetic constraint to the number of cervical vertebrae in mammals, however, suggests that the giraffe has 7 cervical vertebrae [6,7]. Variation in the number of cervicals is associated with an increased incidence of cancers and congenital anomalies [6]. In the present study, we treat the giraffe as a typical mammal with 7 cervical vertebrae.

Darwin classically theorized that the evolutionary elongation of the *Giraffa camelopardalis* neck facilitated browsing on vegetation exceeding heights attainable to co-existing herbivores [8]. Giraffes have been observed to preferentially feed at higher levels, therefore minimizing competition with coexisting browsers [9]. It has also been proposed that sexual selection was the driving force behind the extreme lengthening; male giraffes utilize their massive necks in a specialized form of combat (“necking”), and are consequently favored by estrous females [10,11]. *Okapia johnstoni* also participates in necking for male dominance, however their relatively short cervical vertebrae do not allow for the high-browsing advantage seen in the giraffe [2,12].

The present study provides a comprehensive description of the cervical vertebral features of both extant members of Giraffidae, and compares measurements and anatomic characteristics among the vertebrae of each species. We hypothesize that that there are serial differences in the morphologic patterns of the vertebrae between the giraffe and the okapi.

## Materials and Methods

We compare the anatomy and serial morphology of the cervical vertebrae of *G*. *camelopardalis* and *O*. *johnstoni*, and document distinctive characteristics. The cervical vertebrae used for the comparative morphologic descriptions are derived from the American Museum of Natural History, New York (AMNH), Rijksmuseum of Natural History, Stockholm (RMS) the National Museum of Natural History, Washington D.C. (NMNH), and the Natural History Museum, Basel (NHMBa) mammalogy collections. The *Okapia johnstoni* specimens include: AMNH 51197, AMNH 51904, AMNH 51218, AMNH 51222, AMNH 51198, AMNH 51213, AMNH 51214, AMNH 113802, AMNH 51223, NHMBa 22, and NMNH 399337. The *Giraffa camelopardalis* specimens include: AMNH 82001, AMNH 27666, AMNH 53543, AMNH 27752, RMS 3141, and NMNH 163312. All analyzed specimens are adult animals.

**Fig 1:**
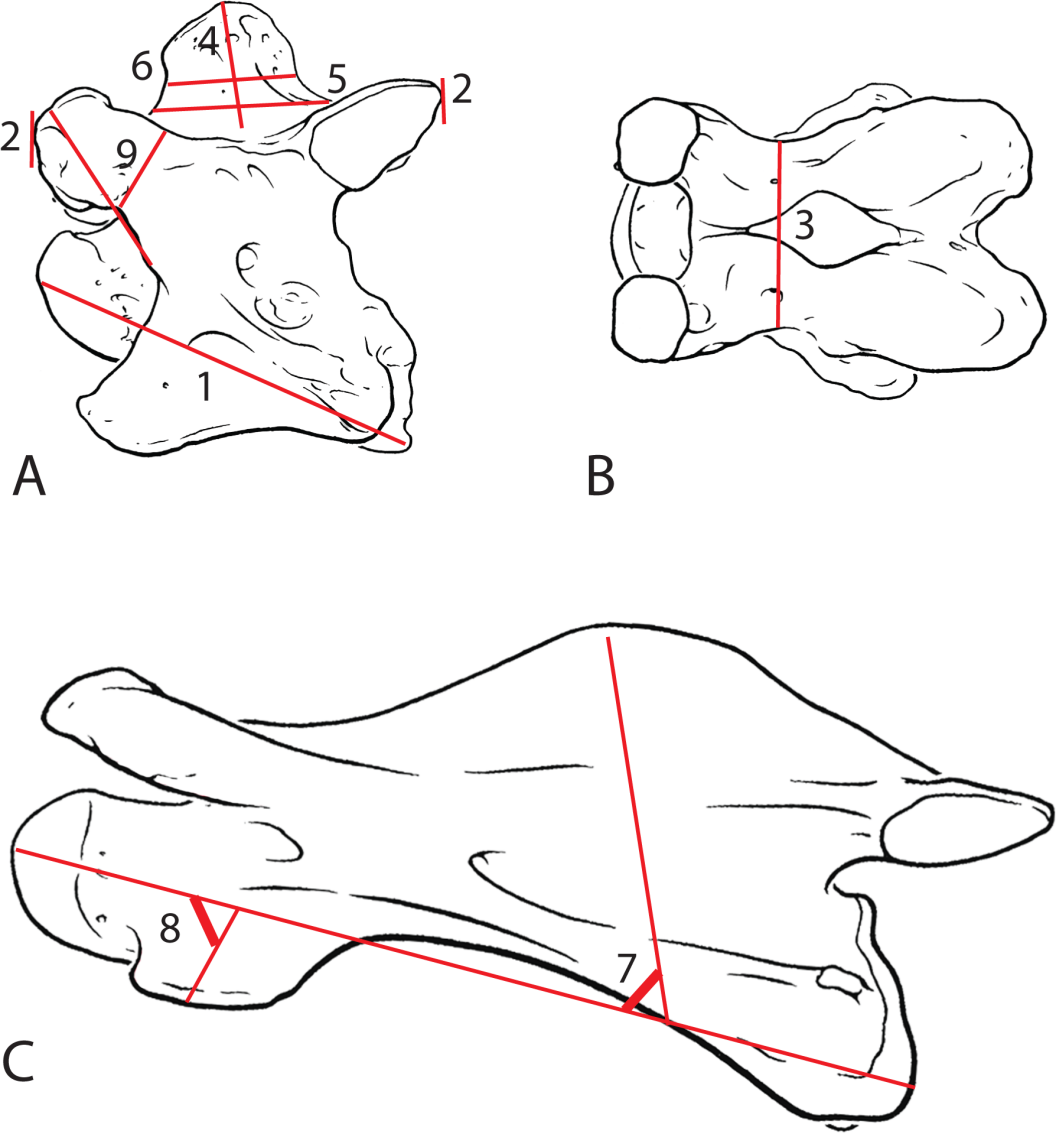
Schematic of measurements used to evaluate giraffid cervical vertebrae. (A) Drawing of the C3 vertebra of *Okapia johnstoni* in lateral view. (B) Drawing of the C3 vertebra of *Okapia johnstoni* in dorsal view. (C) Drawing of the C3 vertebra of *Giraffa camelopardalis* in lateral view. Lines in red represent measurements taken to quantify serial morphologic features. 1- centrum length, 2- maximum length, 3- minimum width, 4- spinous process height, 5- Length of spinous process at base, 6- length of spinous process, 7- angle of spinous process, 8- angle of ventral tubercle, 9- length:width of cranial articular process.

Using three specimens each of *G*. *camelopardalis* and *O*. *johnstoni*, the following measurements were taken (Fig 1). Each measurement was taken with standard calipers in millimeters:

Centrum length: distance between the cranial bulge and the caudal-most point of the vertebral body

Maximum length: distance between the cranial and caudal articular facets

Minimum width: distance between the narrowest points on the dorsal lamina

Height of spinous process: maximum height along the median plane of the spinous process

Length of spinous process at base: distance between the cranial-most and caudal-most aspects of the spinous process on the dorsal lamina

Length of spinous process: distance between the cranial-most and caudal-most aspects of the spinous process on the dorsal lamina 10 mm above the dorsal lamina

Angle of spinous process: angle formed between a median line running through the spinous process and the anterior-posterior axis of the vertebral body

Angle of ventral tubercle: angle formed between a median line running through the ventral tubercle and the anterior-posterior axis of the vertebral body

Length:width ratio of the cranial articular process: distance between the cranial opening of the foramen transversarium and the tip of the cranial articular facet divided by the minimal width of the cranial articular process

We compare the variability of these measurements in C3-C6 of the two taxa in question by calculating the coefficient of variation for each character measured. We exclude C7 from this analysis because the typical C7 vertebra has many morphologic differences from the other cervical vertebrae, and would therefore alter the results.

## Results

### Description of Giraffidae cervical vertebrae (based on *Okapia johnstoni* and *Giraffa camelopardalis*)

**Fig 2:**
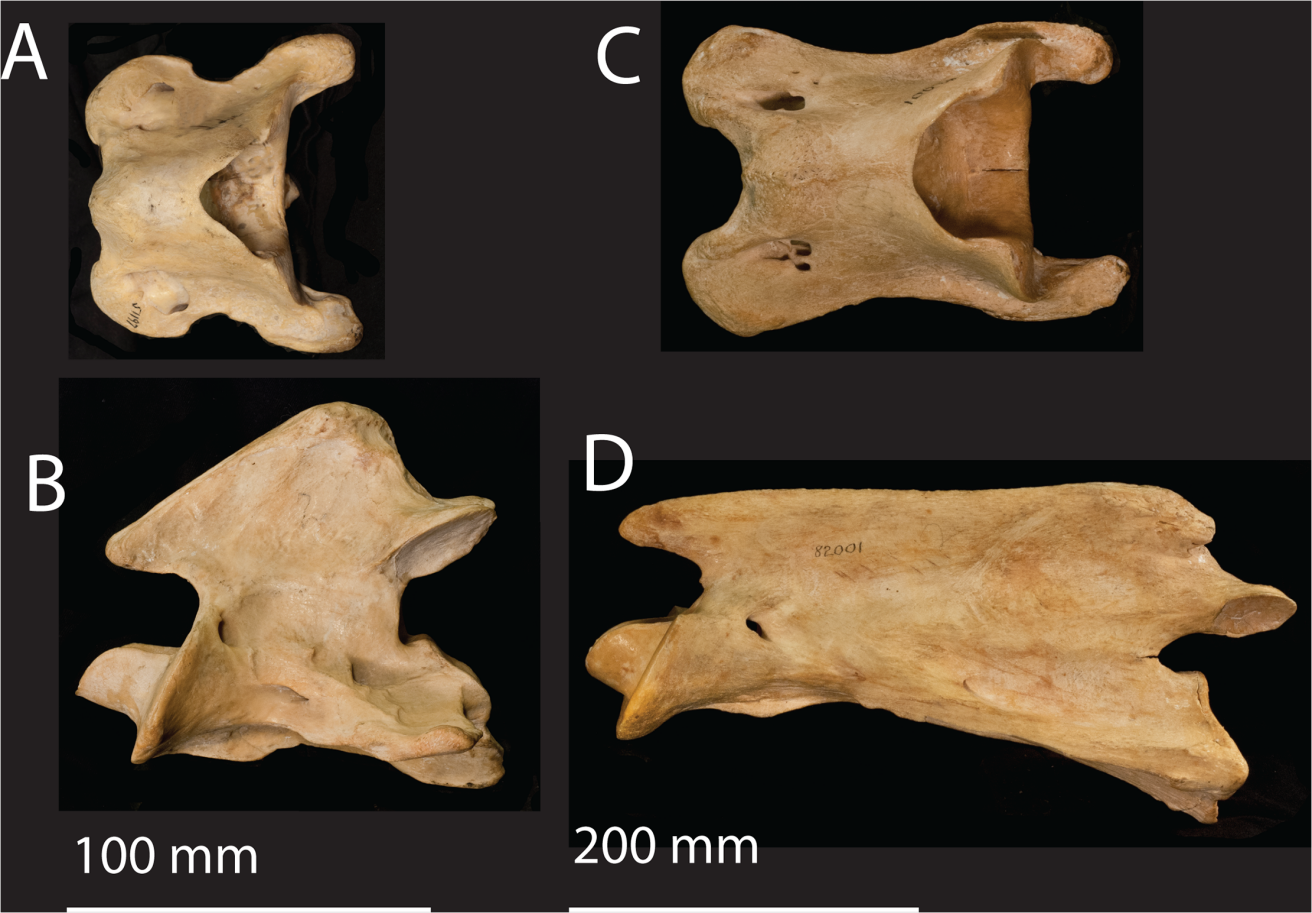
The atlas and axis of the okapi and giraffe. (A) *Okapia johnstoni* (AMNH 51197) atlas in dorsal view. (B) *Okapia johnstoni* axis in ventral view. The scale bar corresponds to 100 mm. (C) *Giraffa camelopardalis* (AMNH 82001) atlas in dorsal view. (D) *Giraffa camelopardalis* axis in ventral view. The scale bar corresponds to 200 mm.

C1: The atlas has a single dorsal and ventral tubercle in the median plane, which are not homologous to the tubercles of the other cervical vertebrae that are laterally positioned and bilateral. The dorsal tubercle is a midline protrusion located in the typical position of a spinous process. The vertebra lacks a pedicle, lamina, and vertebral body, and is instead comprised of an elongated dorsal and ventral arch. The cranial articular facets are strongly concave, and the caudal articular facets are fused at midline. The posterior end of the arches forms a transverse edge dorsally and ventrally. The ventral tubercle is positioned midline and is oriented caudally. (Fig 2)

C2: The axis has a characteristic elongated horizontal spinous process with a notch on the cranial border. The pars interarticularis comprises the border between the lamina and the pedicle. The cranial articular facets form a fused, flattened cranial surface. The odontoid process is dorsally concave, forming a trough with a depression in the center. The cranial surface of the dens is flattened. The vertebra lacks a discrete ventral tubercle. The dorsal tubercle is a distinct protrusion at the caudal vertebral body. (Fig 2)

**Fig 3:**
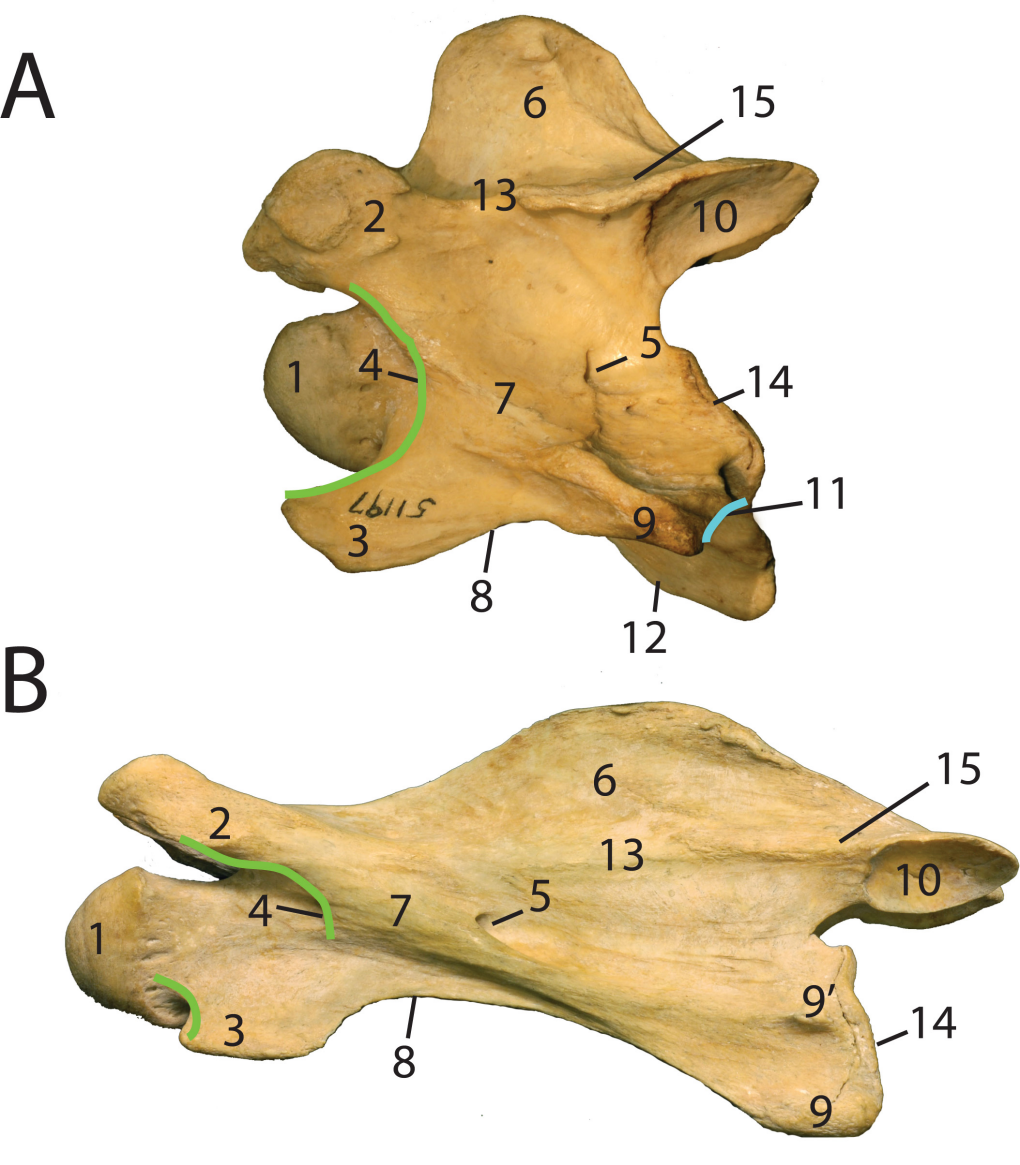
Anatomic Terminology of the giraffe and okapi vertebrae. (A) Labeled C3 vertebra of *Okapia johnstoni* (AMNH 51197). (B) Labeled C3 vertebra of *Giraffa camelopardalis* (AMNH 82001). The anterior arch is drawn in green; *O*. *johnstoni* has a continuous arch, and *G*. *camelopardalis* has an interrupted arch. The post-tubercular ridge of *O*. *johnstoni* is drawn in blue. 1- cranial bulge, 2- cranial articular process, 3- ventral tubercle, 4- cranial opening of the foramen transversarium, 5- caudal opening of the foramen transversarium, 6- spinous process, 7- transverse process, 8- intertubercular plate, 9- dorsal tubercle, 9’-accessory dorsal tubercle, 10- caudal articular facet, 11- post-tubercular ridge, 12- ventral ridge, 13- pars interarticularis, 14- caudal extremity, 15- lamina. [18].

**Fig 4:**
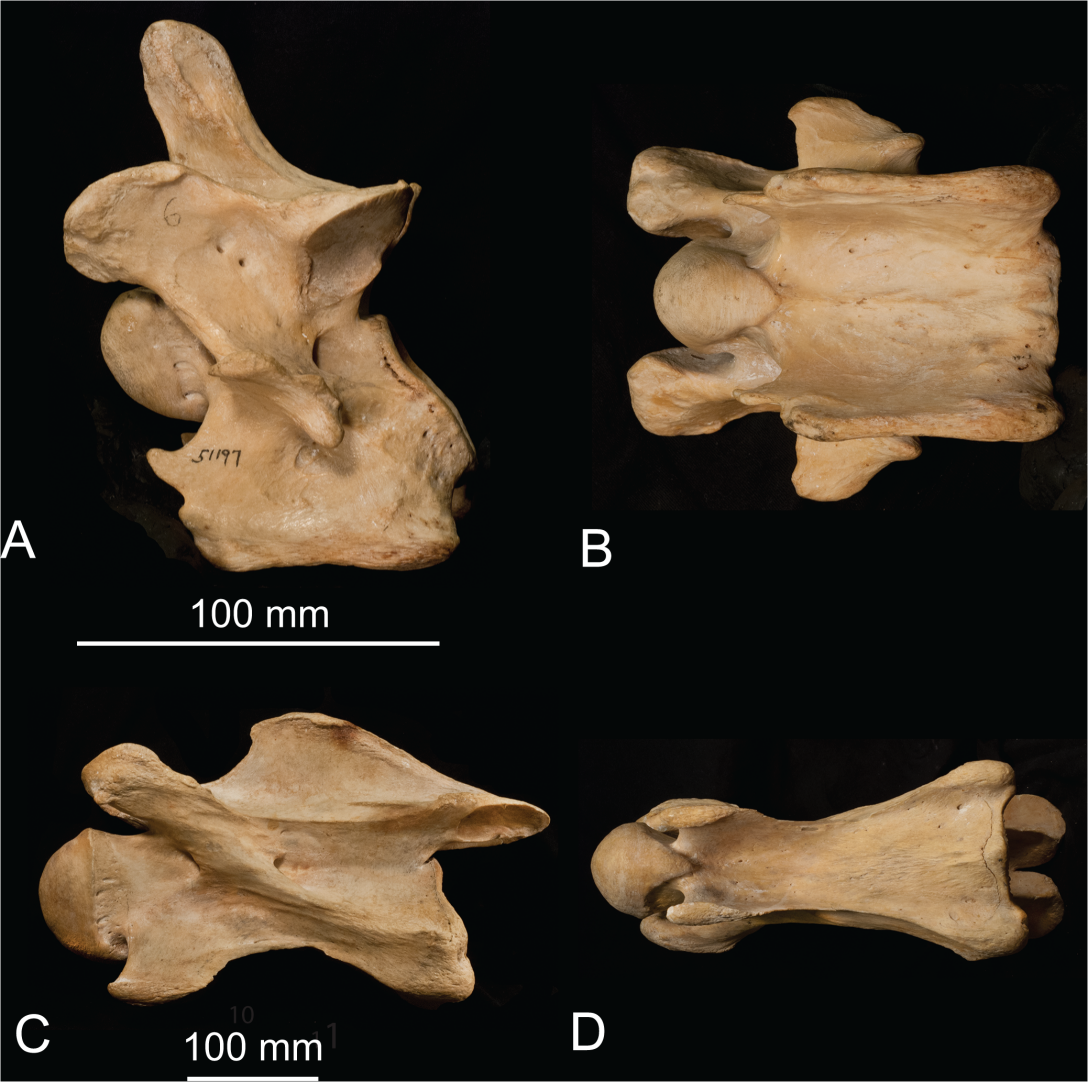
The sixth cervical vertebra of the okapi and giraffe. (A) *Okapia johnstoni* (AMNH 51197) C6 in lateral view. (B) *Okapia johnstoni* C5 in ventral view. The scale bar corresponds to 100 mm. (C) *Giraffa camelopardalis* (AMNH 82001) C6 in lateral view. (D) *Giraffa camelopardalis* C6 in ventral view. The scale bar corresponds to 100 mm.

C3-C7: The base of the spinous process extends over the entire length of the lamina. The spinous process is situated in the center of the dorsal lamina. The lamina is flat and is located above the vertebral body. The pars interarticularis connects the cranial and caudal articular facets, and forms a well-defined edge separating the pedicle from the lamina (Fig 3). In dorsal view, the pars interarticularis is constricted centrally. Dorsally, there is a set of cranial and caudal articular facets that face dorso-medially and ventro-laterally respectively. The cranial and caudal facets connect with the lamina and pedicle via thickened bony material, termed the articular processes. The cranial articular process connects laterally to the transverse process. The transverse process protrudes between the cranial and caudal openings of the foramen transversarium. The cranial opening of the foramen transversarium is set inward, next to a dorso-ventrally oriented arch (termed the anterior arch) (Fig 3). The anterior arch is adjacent to the intervertebral canal where the spinal nerve exits. The vertebral body has a domed cranial end and concave caudal end. The ventral tubercle is cranial to the transverse process, and the dorsal tubercle is caudal to the transverse process. C6 in the okapi has an expanded plate termed the ventral lamina (Fig 4). C7 has several specializations that differ in the giraffe and the okapi (see description below).

### Representative measurements of *Okapia johnstoni* and *Giraffa camelopardalis* vertebrae

Tables 1 and 2 show measurements and vertebral characters of the two giraffid taxa to quantify the serial morphology of the cervical vertebrae. The coefficient of variation for the C3 to C6 vertebrae is smaller in *G*. *camelopardalis* than an in *O*. *johnstoni* in the following characters: centrum length, maximum length, minimal width, spinous process height, length of spinous process at base, length of spinous process, angle of ventral tubercle, and length:width of cranial articular process (Table 3). The coefficient of variation for the angle of the spinous process is smaller in *O*. *johnstoni*.

**Table 1:** Selected measurements of *Giraffa camelopardalis* C1-T1 vertebrae.

Specimen #	Vertebra	Centrum length	Maximum length	Minimum width	Spinous process height	Length of spinous process at base	Length of spinous process	Angle of spinous process	Angle of ventral tubercle	L:W of cranial articular process
AMNH 27666	C1	139.58	169.14	82.23						
AMNH 27752	C1	144.8	173.01	105.03						
AMNH 82001	C1	150.56	184.61	101.06						
AMNH 27666	C2	314	330	34.26				164		
AMNH 27752	C2	309	323	51.49				162		
AMNH 82001	C2	297.99	326.37	31.86				160		
AMNH 27666	C3	308	337	37.63	46.14	233	175.25	110	82	4.01
AMNH 27752	C3	295	353	45.64	53.59	209.24	117.19	107	80	3.28
AMNH 82001	C3	310	350	34.11	51.49	230.68	164.25	104	78	3.44
AMNH 27666	C4	306	345	35.88	41.45	242.71	209.28	117	88	3.6
AMNH 27752	C4	310	347	44.74	44.47	261.01	186.43	98	85	3.71
AMNH 82001	C4	297	342	38.32	48.29	216.69	157.89	93	84	2.92
AMNH 27666	C5	292	336	41.11	54.28	247.7	197.82	92	87	3.56
AMNH 27752	C5	297	347	48.82	55.73	244.26	203.23	90	85	3.33
AMNH 82001	C5	293	341	43.33	50.92	230.35	163.83	93	86	3.08
AMNH 27666	C6	280	304	49.55	62.78	214.57	174.81	81	81	3.41
AMNH 27752	C6	292	316	58.36	56.82	196.24	164.69	62	50	2.21
AMNH 82001	C6	284	315	49.3	59.93	218.2	149.48	95	72	2.87
AMNH 27666	C7	252	230	62.12	114.22	103.99	88.16	67	83	3.03
AMNH 27752	C7	260.61	240.19	66.44	108.11	98.4	91.69	63	81	2.05
AMNH 82001	C7	271.79	236.43	68.26	96.32	89.61	59.61	63	69	2.69
AMNH 27752	T1	127.11	152.31	69.41	295	78.22	72.27	82		1.61
AMNH 82001	T1	157.19	180.17	68.48	144.44	76.56	50.26	62		

**Table 2:** Selected measurements of *Okapia johnstoni* C1-T1 vertebrae.

Specimen #	Vertebra	Centrum length	Maximum length	Minimum width	Spinous process height	Length of spinous process at base	Length of spinous process	Angle of spinous process	Angle of ventral tubercle	L:W of cranial articular process
AMNH 51197	C1	68.12	90.49	75.83						
AMNH 51198	C1	68.83	96.55	89.15						
AMNH 51213	C1	68.1	93.23	98.16						
AMNH 51197	C2	98	121.22	41.18				112		
AMNH 51198	C2	98.63	118.02	30.66				130		
AMNH 51213	C2	100.29	129.34	40.6				131		
AMNH 51197	C3	98.8	105.51	43.18	41.69	61.86	45.93	79	49	1.39
AMNH 51198	C3	99.65	104.07	43.49	33.86	52.13	38.77	77	55	2.01
AMNH 51213	C3	102.45	106.68	52.81	29.86	56.38	40.27	69	37	2.21
AMNH 51197	C4	103.03	106.73	58.92	39.55	55.39	43.16	72	66	1.38
AMNH 51198	C4	100.66	106.07	60.03	34.81	49.02	34.02	65	58	1.91
AMNH 51213	C4	109.16	102.83	65.05	35.1	44.71	34.78	75	44	1.84
AMNH 51197	C5	107.97	102.2	66.96	48.07	50.42	33.56	67	76	1.34
AMNH 51198	C5	101.07	100.43	68.71	41.3	41.31	28.28	66	58	1.76
AMNH 51213	C5	107.07	101.9	70.07	42.46	40.73	27.22	66	45	1.69
AMNH 51197	C6	107.4	92.13	67.22	60.42	42.58	32.3	58		1.51
AMNH 51198	C6	103.36	93.72	70.88	48.9	41.47	26.94	57		1.99
AMNH 51213	C6	111.02	97.91	73.24	45.96	40.85	28.03	54		1.89
AMNH 51197	C7	92.54	89.98	64.73	85.18	40.66	30.23	66		
AMNH 51198	C7	87.26	86.3	64.09	89.7	42.98	35.82	60		
AMNH 51213	C7	83.17	94.6	68.64	84.79	47.34	33.45	59		
AMNH 51197	T1	64.28	81.92	35.82	142.05	48.23	45.51	100		
AMNH 51198	T1	52.33	81.56	37.37	147.64	46.49	41.11	82		
AMNH 51213	T1	63.2	81.4	34.08	157.83	39.69	36.69	80		

**Table 3:** Coefficient of variation of C3-C6 for eleven measurements in *G*. *camelopardalis* and *O*. *johnstoni*.

	*G*. *camelopardalis*	*O*. *johnstoni*
**Centrum length**	3.31	3.89
**Maximum length**	4.69	4.8
**Minimum width**	15.91	16.71
**Spinous process height**	12.14	19.85
**Length of spinous process at base**	8.08	15.01
**Length of spinous process**	14.82	18.65
**Angle of spinous process**	15.1	11.8
**Angle of ventral tubercle**	13.02	22.17
**Length:width of cranial articular process**	14.3	16.27

### Serial cervical vertebral morphologic variations between *Okapia johnstoni* and *Giraffa camelopardalis*

#### Spinous process

**Fig 5:**
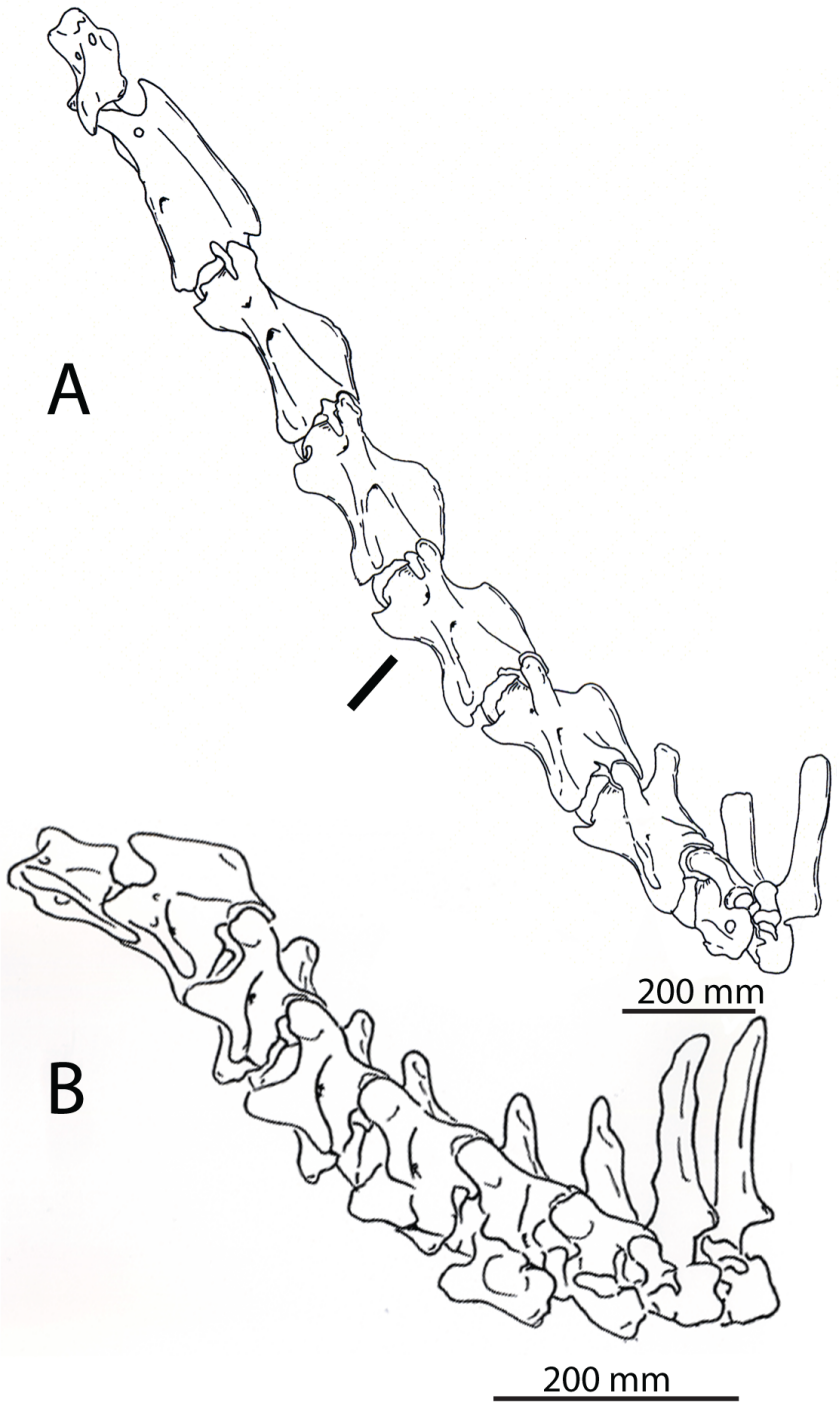
Complete articulated necks of the giraffe and the okapi. (A) Lateral view of C1-T2 of *Giraffa camelopardalis* (AMNH 82001). (B) Lateral view of C1-T2 of *Okapia johnstoni* (NMNH 399337). The black line demarcates the actual length of the *O*. *johnstoni* neck in relation to that of *G*. *camelopardalis*. The scale bar corresponds to 200 mm.

**Fig 6:**
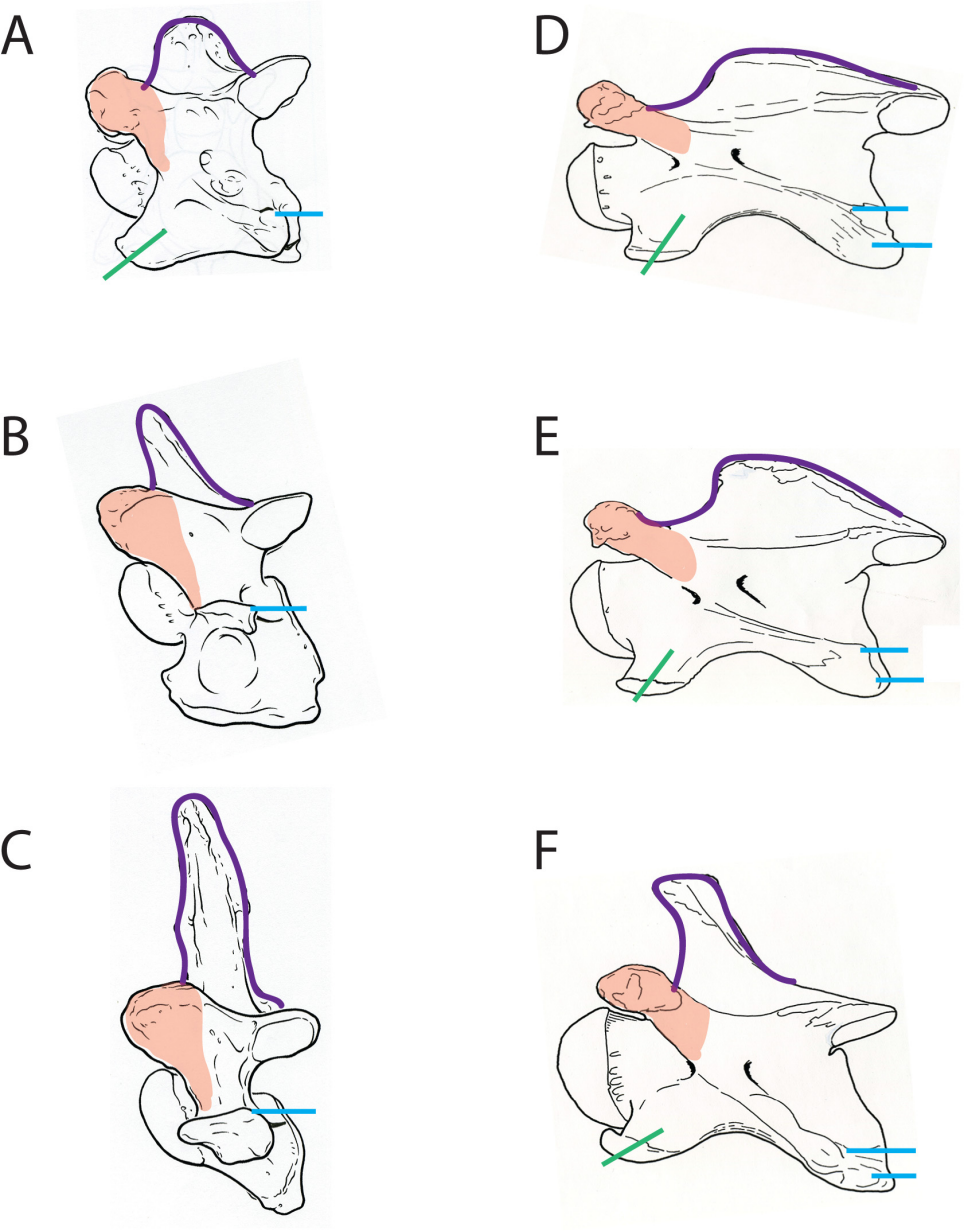
Lateral views of the okapi and giraffe vertebrae showing morphologic characters. (A) Lateral view of *Okapia johnstoni* C5 vertebra. (B) Lateral view of *Okapia johnstoni* C6 vertebra. (C) Lateral view of *Okapia johnstoni* C7 vertebra. (D) Lateral view of *Giraffa camelopardalis* C5 vertebra. (E) Lateral view of *Giraffa camelopardalis* C6 vertebra. (F) Lateral view of *Giraffa camelopardalis* C7 vertebra. The outline of the spinous process is drawn in purple, the cranial articular process is highlighted in orange, the position of the dorsal tubercle is marked in blue, and the orientation of the ventral tubercle is drawn in green. The *Okapia johnstoni* C6 and C7 vertebrae lack a ventral tubercle (typical of ruminants). The characters drawn suggest homogenization of the *G*. *camelopardalis* cervical vertebrae, relative to *O*. *johnstoni*.

In the okapi, the base of the spinous process comprises the length of the dorsal lamina. It presents as an elongated protrusion expanding the length of the lamina on C3-C4, and becomes more confined and elongated from C5 to C7 (Fig 5B). It is progressively oriented more rostrally from C3 to C7 (Fig 6A, 6B, and 6C). It is situated cranially, positioned at the base between the cranial articular processes. The surface of the spinous process of C6 is textured, with several longitudinal ridges that continue onto the caudal aspect of the dorsal lamina.

In the giraffe, like the okapi, the spinous process comprises the majority of the dorsal vertebral body. It presents as an elongated protrusion whose base expands the entire length of the lamina on C3-C6 (Fig 6D and 6E). On C7, the spinous process becomes a more confined, elongated protrusion (Fig 6F). The orientation is relatively uniform as slightly rostral from C3-C6, and is more rostral on C7. The spinous process is situated caudally on C3-C5, and becomes more central on C6-C7.

#### Anterior arch and cranial articular process

In the okapi, the anterior arch is uninterrupted (Fig 3A). The anterior arch is a ridge that connects the cranial articular process to the ventral tubercle. The continuity of the anterior arch is most obvious from C3-C4. The cranial opening of the foramen transversarium is situated anterior to this ridge. The base of the cranial articular process becomes progressively more expanded from C3-C7 (Fig 6A, 6B and 6C). In dorsal view, the cranial articular facet attaches directly to the lamina.

In the giraffe, the ridge forming anterior arch is interrupted (Fig 3B). The neck and vertebral body are lengthened cranial to the arch, and the cranial articular process is displaced and disconnected from the ridge caudally. There is therefore no ridge connecting the cranial articular facet with the ventral tubercle. The base of the cranial articular process remains narrow and tubular from C3-C7 (Fig 6D, 6E and 6F). In dorsal view, the cranial articular facet is disconnected with the lamina by a longer articular process.

#### Dorsal tubercle

In the okapi, the dorsal tubercle is aligned with the ventral tubercle in C3-C5, and is a dorsal thickening at the caudal vertebral body. It connects with the ventral tubercle by a thin, elevated bony ridge termed the intertubercular plate. An elevated ridge connects the midpoint of the caudal vertebral body to the posterior aspect of the dorsal tubercle (Fig 3A). We term this the post-tubercular ridge (new term). This ridge defines the ventral border of the caudal opening of the foramen transversarium. In C6-C7, the dorsal tubercle changes position from the previous vertebrae, and realigns directly caudal to the transverse process (Fig 6A and 6B). The post-tubercular is present regardless of the position of the dorsal tubercle. The dorsal tubercle attaches to the transverse process by a bony ridge, which becomes progressively shorter as the two protrusions approximate.

In the giraffe, the dorsal tubercle remains in the same plane as the ventral tubercle throughout C2-C7 (Fig 6D, 6E and 6F). The dorsal tubercle presents as two discrete protrusions; we term more dorsal protrusion the accessory dorsal tubercle (Fig 3B). Both the dorsal tubercle and the accessory dorsal tubercle are caudal thickenings of bony material at the caudal margin of the vertebral body. The dorsal tubercle connects to the ventral tubercle with an excavated intertubercular bony plate. This thin plate has an expanded notch in lateral view. The accessory dorsal tubercle connects to the transverse process with an elevated bony ridge. This ridge becomes progressively more prominent from C3 to C7.

#### Ventral tubercle

In the okapi, the ventral tubercle of C3-5 forms a wide rostral protrusion that connects with the dorsal tubercle. The ventral tubercle of C3 is strongly oriented rostrally, and re-orients progressively more caudally on C4-C5. In C6, there is an expanded ventral plate, termed the ventral lamina (Fig 4). The ventral lamina is antero-posteriorly directed and the ventral edge of this plate is uniformly thickened. In lateral view, the ventral lamina has a central, circular fossa. There is no distinct ventral tubercle in C6 or C7.

In the giraffe, the ventral tubercle presents as an expanded protrusion cranially throughout C3-C7 (Fig 5A). The orientation of the ventral tubercle is uniform in C3-C5 as ventral, and re-orients slightly more rostrally in C6-C7. There is no expansion of the ventral lamina into a plate on C6; the dorsal and ventral tubercles persist as discrete, separated protrusions on the ventral vertebral body (Fig 6D, 6E and 6F). The ventral tubercle is approximately equal-sized in C3-C6, and is slightly shortened, but still present in C7.

#### Shape of ventral atlas, vertebral body and ventral ridge

**Fig 7:**
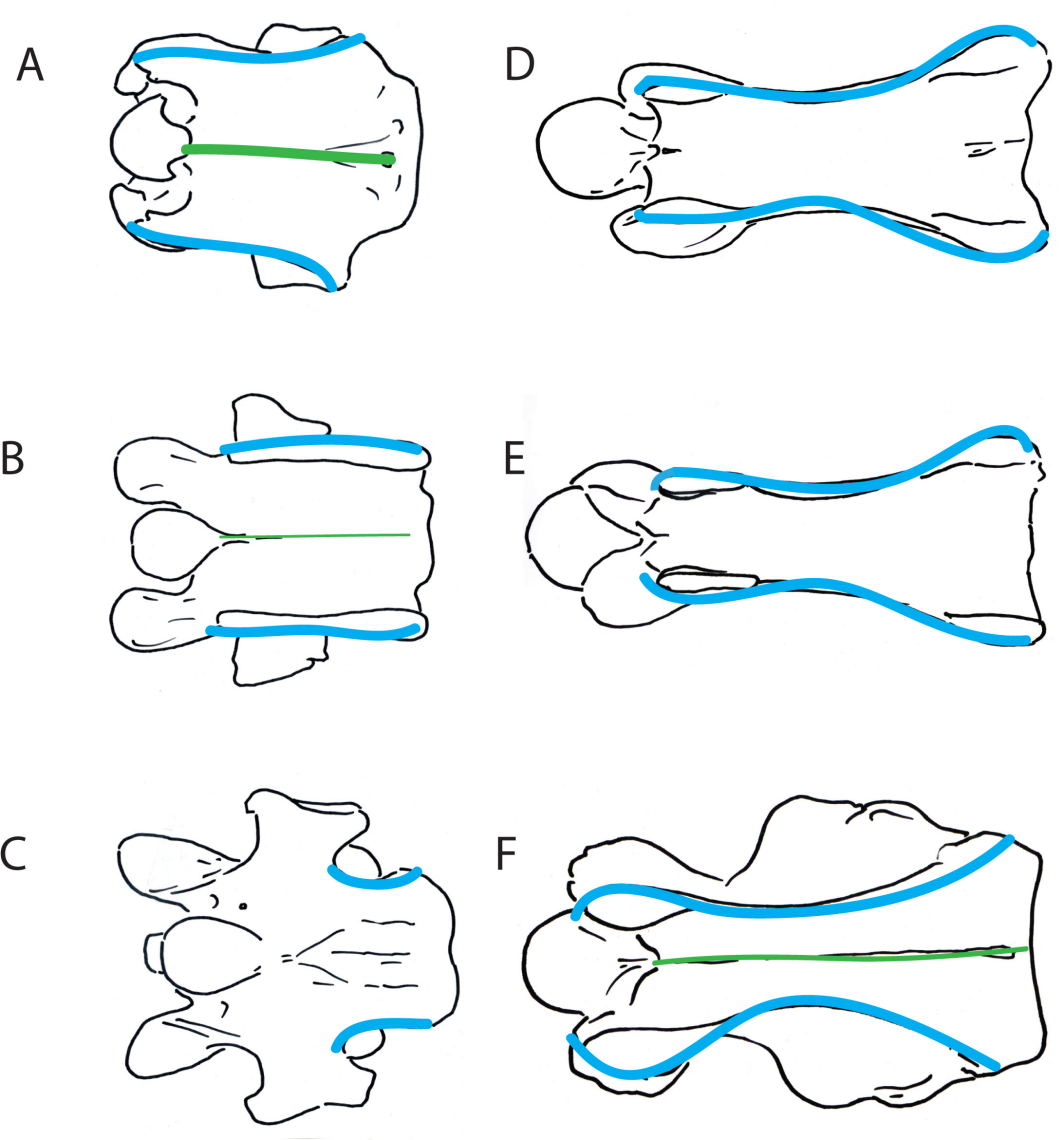
Ventral views of okapi and giraffe vertebrae showing morphologic characters. (A) Ventral view of *Okapia johnstoni* C5 vertebra. (B) Ventral view of *Okapia johnstoni* C6 vertebra. (C) Ventral view of *Okapia johnstoni* C7 vertebra. (D) Ventral view of *Giraffa camelopardalis* C5 vertebra. (E) Ventral view of *Giraffa camelopardalis* C6 vertebra. (F) Ventral view of *Giraffa camelopardalis* C7 vertebra. The shape of the ventral vertebral body and intertubercular plate is shown in blue, and the ventral ridge, when present, is drawn in green.

The ventral lamina of the atlas of the okapi is short, and laterally has a medial constriction. The length of the dorsal and ventral arches is approximately equal to that of the odontoid process of the axis. In the okapi, the shape of the vertebral body in ventral view varies throughout the cervical vertebrae. In C2, the cranial and caudal widths are relatively equal, and there is a deep constriction slightly rostral to the center. In C3-C5, the ventral vertebral body and intertubercular plate is rhomboidal-shaped, where the caudal edge is more laterally expanded than the rostral aspect (Fig 7A). In C6, the ventral vertebral body is square-shaped, with the lateral edges parallel to one another (Fig 7B). In C7, the ventral vertebral body itself is rectangular-shaped, and the transverse processes protrude laterally at the center (Fig 7C). From C2-C5, there is a distinct ventral ridge at midline, which is continuous longitudinally on the vertebral body. On C6, the ventral ridge is very faint.

In the giraffe, the dorsal and ventral arches of the atlas are elongated, and exceed the length of the odontoid process of the axis. The shape of the ventral vertebral body is relatively uniform from C2-C7. In ventral view, the vertebral body and intertubercular plate is hourglass shaped, where the plate connecting the ventral and dorsal tubercles is constricted centrally (Fig 7D, 7E and 7F). The caudal edge is slightly more expanded than the cranial edge. There is a discontinuous, faint ventral ridge on C3, and the ridge is absent on C4-C6. On C7, there is a faint ventral ridge that is continuous longitudinally on the ventral vertebral body.

## Discussion

The ruminant neck has, in addition to other minor functions, a dual role in both feeding and intra-specific male fighting, however osteologic studies of cervical vertebrae to correlate to function are lacking. The elongated neck of the giraffe has functional implications, including high-browsing and male combat [9, 13]. The giraffe neck also swings to change the center of gravity of the skull during locomotion, facilitating fluid body movements [14]. The okapi has also been observed to utilize their neck during fighting, however this phenomenon has not been researched in detail [12]. Dissimilarities in the serial anatomic patterns of the giraffe and okapi cervical vertebrae suggest potential differences in the specific use of the neck. The structure of the giraffe cervical vertebrae enable feeding both at shoulder level and at high levels up to five meters [10,15]. The giraffe cervical vertebrae exhibit substantial vertebral lengthening exceeding that of the okapi, which allows for high browsing as well as feeding from lower levels [2]. This cervical elongation and resulting structural vertebral changes seen in the giraffe permits a greater range in feeding not seen in the okapi.

The lengthening of the giraffe cervical vertebrae perceptibly exceeds that of any living ungulate, including the other extant giraffid [2]. Our morphologic descriptions of the serial cervical vertebrae demonstrate that there is considerable homogenization of the *G*. *camelopardalis* cervicals which is not seen in *O*. *johnstoni*. In the giraffe, the position of the dorsal tubercle, thickness of the cranial articular process, orientation of the ventral tubercle, and hourglass ventral shape are uniform throughout C3-C7, whereas in the okapi, these features are serially variable. Correspondingly, the coefficient of variation of C3-C6 for ten of the eleven measurements is smaller in *G*. *camelopardalis*, supporting serial homogenization. We believe this homogenization results from the extreme cervical elongation seen only in the giraffe.

The locus of the dorsal tubercle is not inherently obvious when observing a cervical vertebra. In the giraffe, the position of the dorsal tubercle is uniform throughout C3-C7, however the position changes in the okapi vertebrae. We utilize the post-tubercular ridge to confirm the position of the dorsal tubercle in the okapi cervical vertebrae. Using this feature, we find that the dorsal tubercle is aligned with the ventral tubercle in C3-C5, and re-orients to situate directly posterior to the transverse process in C6-C7. In the giraffe, the dorsal tubercle remains aligned with the ventral tubercle in C3-C7, further contributing to the serial homogenization. Unlike other ruminants, the giraffe has an additional accessory dorsal tubercle and both are caudally positioned, suggesting functional changes of the vertebrae. We believe the double dorsal tubercle of the giraffe increases the area for muscle attachment, therefore providing additional support for the massive neck.

The okapi C6, like in other ruminants, possesses a plate in the position of the ventral tubercle termed the ventral lamina. We find this plate has a circular fossa centrally, most visible in lateral view. The giraffe C6 more closely resembles the preceding cervical vertebrae, and instead of the ventral plate, possesses widely spaced ventral and dorsal tubercles, connected by a thin, concave ridge of bone. We believe this concavity can be explained by an allometric stretching of the vertebral body, further displacing the ventral tubercle in relation to the dorsal tubercle, and thinning the bony connection between the two protrusions.

The positions of the articular facets differ in the ruminant cervical and thoracic vertebrae. In the cervicals, the cranial articular facet is located at the end of the cranial articular process, and the caudal articular facet is situated on the caudal edge of the lamina. In the thoracic region, however, the cranial articular facet is located on the lamina and the caudal articular facet medially on the inferior base of the spinous processes [2]. In dorsal view, the cervical articular facets are positioned laterally, and those in the thoracic vertebrae are medially approximated. At the typical cervico-thoracic junction, T1 is structured to connect the wide cervical facets to the narrow thoracic facets [1]. The first thoracic vertebra therefore necessitates a combination of cervical and thoracic morphology with widely spaced cranial facets and narrow caudal facets. The giraffe is the only known mammal where the transitional vertebra containing wide cranial articular facets and narrow caudal articular facets is T2; the T1 resembles cervical vertebrae with both sets of facets being wide [3, 16]. This specialization is best seen in the juvenile giraffe T1 vertebra. In the adult giraffe T1, there is a slight narrowing on the posterior side of the vertebra, but it is still significantly wider and longer than that of a typical thoracic. This specialization is not seen in the okapi, whose cervico-thoracic junction is typical of a ruminant [3].

Lankester proposed a “cervicalized” role of the giraffe T1 and subsequently Solounias provided evidence that the first thoracic vertebra functions similar to a typical ruminant C7 [1, 3]. Notably, this giraffe vertebra possesses a dorsal and ventral tubercle, a functional foramen transversarium, and an elongated and flat transverse process. The roots of the brachial plexus further substantiate these osteologic observations; they exit one vertebral level caudal than in the okapi and other ruminants [3]. In accordance with these results, we find the C7 of the giraffe shares many characteristics of the preceding cervical vertebrae, including the position and orientation of the spinous process and ventral and dorsal tubercles, as well as the vertebral body shape. In the okapi, however, the C7 vertebra is uniquely structured compared with the previous vertebrae, with a realigned dorsal tubercle, more prominent post-tubercular ridge, and boxy-shaped vertebral body. We believe these vertebral modifications allow the T1 of the giraffe to function as the C7, and therefore provide the greatest support for the entire length of the neck. The T1 vertebra presumably possesses additional structural support from the thoracic ribs and muscles. This is especially beneficial in the giraffe, whose neck comprises a substantial portion of total body mass [17].

Osteologic comparisons of the giraffe and okapi cervical vertebrae demonstrate differing serial anatomic patterns. We find the giraffe to be substantially more homogenized in morphology between the vertebrae, whereas the okapi have several characteristics that are variable from vertebra to vertebra. These structural differences presumably have implications on the function and adaptation of the okapi and giraffe neck.
